# Characteristic of COVID-19 infection in pediatric patients: early findings from two Italian Pediatric Research Networks

**DOI:** 10.1007/s00431-020-03683-8

**Published:** 2020-06-03

**Authors:** Niccolò Parri, Anna Maria Magistà, Federico Marchetti, Barbara Cantoni, Alberto Arrighini, Marta Romanengo, Enrico Felici, Antonio Urbino, Liviana Da Dalt, Lucio Verdoni, Benedetta Armocida, Benedetta Covi, Ilaria Mariani, Roberta Giacchero, Anna Maria Musolino, Marco Binotti, Paolo Biban, Silvia Fasoli, Chiara Pilotto, Flavia Nicoloso, Massimiliano Raggi, Elisabetta Miorin, Danilo Buonsenso, Massimo Chiossi, Rino Agostiniani, Anna Plebani, Maria Antonietta Barbieri, Marcello Lanari, Serena Arrigo, Elena Zoia, Matteo Lenge, Stefano Masi, Egidio Barbi, Marzia Lazzerini, on behalf of the CONFIDENCE and COVID-19 Italian Pediatric Study Networks

**Affiliations:** 1 Department of Emergency Medicine and Trauma Center, Meyer University Children’s Hospital, Florence, Italy; 2 Department of Pediatrics, Community Pediatrics, Ravenna, Italy; 3 Department of Pediatrics, Ravenna Hospital, Ravenna, Italy; 4 Healthcare Professional Department Fondazione IRCCS Ca’ Granda, Ospedale Maggiore Policlinico, Milan, Italy; 5 Pediatric Emergency Department, Presidio Ospedale dei Bambini, ASST Spedali Civili, Brescia, Italy; 6 IRCCS Istituto Gaslini, Genoa, Italy; 7 Pediatric and Pediatric Emergency Unit, The Children Hospital, Azienda Ospedaliera SS Antonio e Biagio e Cesare Arrigo, Alessandria, Italy; 8 Department of Pediatric Emergency, Regina Margherita Children’s Hospital - A.O.U. Città della Salute e della Scienza di Torino, Turin, Italy; 9 Department for Woman and Child Health-Pediatric Emergency Department, University of Padua, Padua, Italy; 10 Department of Pediatrics, Papa Giovanni XXIII Hospital, Bergamo, Italy; 11 Institute for Maternal and Child Health - IRCCS “Burlo Garofolo”, Trieste, Italy; 12 Department of Pediatrics, Lodi Hospital, Lodi, Italy; 13 Department of Pediatric Emergency Medicine, Bambino Gesù Children’s Hospital, IRCCS, Rome, Italy; 14 Neonatal and Pediatric Intensive Care Unit, Maggiore della Carità University Hospital, Novara, Italy; 15 Department of Neonatal and Paediatric Critical Care, Verona University Hospital, Verona, Italy; 16 Paediatric Unit, Carlo Poma Hospital, Mantua, Italy; 17 Division of Paediatrics, Department of Medicine DAME, Academic Hospital Santa Maria della Misericordia, University of Udine, Udine, Italy; 18 Family Pediatrician, Udine, Italy; 19 ICU, Pain Therapy Unit, Rovereto Hospital, Trento, Italy; 20 Department of Pediatrics, Latisana-Palmanova, ASUFC, Udine, Italy; 21 Department of Woman and Child Health and Public Health, Fondazione Policlinico Universitario A. Gemelli IRCCS, Rome, Italy; 22 Università Cattolica del Sacro Cuore, Rome, Italy; 23 Department of Pediatrics, ASL 4 Liguria, Lavagna, Italy; 24 Department of Pediatrics, Ospedale San Jacopo, Pistoia, Italy; 25 Pediatric Emergency Unit, Filippo Del Ponte Hospital, ASST-Settelaghi, Varese, Italy; 26 Pediatric Emergency Unit, S. Orsola Hospital, University of Bologna, Bologna, Italy; 27 Department of Pediatrics, Hospital Filippo Del Ponte, Varese, Italy; 28 Department of Pediatrics, Hospital V. Buzzi, Milan, Italy; 29 Clinical Trial Office, Children’s Hospital A. Meyer-University of Florence, Florence, Italy; 30 Child Neurology Unit and Laboratories, Neuroscience Department, Children’s Hospital A. Meyer-University of Florence, Florence, Italy; 31 Functional and Epilepsy Neurosurgery Unit, Neurosurgery Department, Children’s Hospital A. Meyer-University of Florence, Florence, Italy; 32 Department of Medicine, Surgery and Health Science, Department of Pediatrics, University of Trieste, Trieste, Italy

**Keywords:** COVID-19, Children, Adolescents, Italy

## Abstract

Detailed data on clinical presentations and outcomes of children with COVID-19 in Europe are still lacking. In this descriptive study, we report on 130 children with confirmed COVID-19 diagnosed by 28 centers (mostly hospitals), in 10 regions in Italy, during the first months of the pandemic. Among these, 67 (51.5%) had a relative with COVID-19 while 34 (26.2%) had comorbidities, with the most frequent being respiratory, cardiac, or neuromuscular chronic diseases. Overall, 98 (75.4%) had an asymptomatic or mild disease, 11 (8.5%) had moderate disease, 11 (8.5%) had a severe disease, and 9 (6.9%) had a critical presentation with infants below 6 months having significantly increased risk of critical disease severity (OR 5.6, 95% CI 1.3 to 29.1). Seventy-five (57.7%) children were hospitalized, 15 (11.5%) needed some respiratory support, and nine (6.9%) were treated in an intensive care unit. All recovered.

*Conclusion*:This descriptive case series of children with COVID-19, mostly encompassing of cases enrolled at hospital level, suggest that COVID-19 may have a non-negligible rate of severe presentations in selected pediatric populations with a relatively high rates of comorbidities. More studies are needed to further understand the presentation and outcomes of children with COVID-19 in children with special needs.

**Table:** 

**What is Known:** *• There is limited evidence on the clinical presentation and outcomes of children with COVID-19 in Europe, and almost no evidence on characteristics and risk factors of severe cases.*	
**What is New:** • *Among a case series of 130 children, mostly diagnosed at hospital level, and with a relatively high rate (26.2%) of comorbidities, about three-quarter had an asymptomatic or mild disease.* *• However, 57.7% were hospitalized, 11.5% needed some respiratory support, and 6.9% were treated in an intensive care unit.*

**Electronic supplementary material:**

The online version of this article (10.1007/s00431-020-03683-8) contains supplementary material, which is available to authorized users.

## Background

The worldwide outbreak of a new type of coronavirus disease (COVID-19) originated in Wuhan, China, in December 2019 and has rapidly spread in most countries in the world, despite governments’ containment measures trying to minimize impact [1, 2]. However, despite global spread, the full clinical spectrum and epidemiological features of COVID-19, particularly in children, are still poorly described [3]. The largest Chinese case series included 2143 children, but of these, only 34.1% (731) were laboratory-confirmed [4]. Very few studies described COVID-19 among children in countries outside China. So far, only two contributes, in the form of research letters, on COVID-19 case series among children from European countries—specifically, Italy and Spain—have been released, and they included small samples and limited details on children characteristics [5, 6]. Although preliminary surveillance data on COVID-19 pediatric cases in the USA has been published, information on clinical presentation was available only in 9% of cases [7]. In general, data from the national surveillance systems [7–9] often miss details on key clinical characteristics of children and their outcomes.

In this retrospective study, we aimed at describing the clinical presentation, diagnostic findings, type of respiratory support, and outcomes of a cohort of pediatric patients with confirmed COVID-19 virus infection in Italy, collected through two large collaborative research networks.

## Methods

### Population and settings

Data were collected through two large collaborative research networks, including a group of pediatric Emergency Departments coordinated by Meyer Hospital in Florence, and a research network of pediatric hospitals/departments and family pediatricians, coordinated by the Institute for Maternal and Child Health IRCCS Burlo Garofolo, Trieste, Italy. Overall, the two networks comprised 61 centers: 53 (86.9%) hospitals and 8 (13.1%) outpatient centers. All children (aged 0–18 years) who presented to any of the recruiting centers between the 3rd and 26th of March 2020 and were diagnosed with COVID-19 were included in the study. Only three of the cases reported within the research network, all referred with very mild disease, could not be retrieved due to unavailability (sick leave) of the doctor who took in charge of them.

Cases were screened for COVID-19 virus infection based on national recommendations during the study period [10]. COVID-19 virus infection was diagnosed using nasal or nasopharyngeal swab specimens collected by trained personnel in line with national recommendations and tested for COVID-19 virus nucleic acid in regional referral laboratories using WHO-recommended real-time reverse transcriptase polymerase chain reaction (RT-PCR) assays.

### Data collection

Data were collected with a predefined, standardized, field-tested form. Clinical, laboratory, and imaging data were obtained from official medical records and entered in the form by staff at each hospital. Information for health workers on how to complete the form was embedded in the form itself. Data collection forms were checked in real time for internal consistency or missing data by trained personnel. Additional cross-check and data cleaning were done before data analysis, by an expert statistician (IM). Disease severity was classified adapting a previous published classification [4], based on predefined criteria, as reported in Table 1**.**

**Table 1:** Disease severity

**Asymptomatic**: all the following must be present	
1. No signs or symptoms	
2. AND negative chest X-ray	
3. AND absence of criteria for other cases	
**Mild**: any of the following (AND absence of criteria for more severe cases)	
1. Symptoms of upper respiratory tract infection	
2. AND absence of pneumonia at chest X-ray	
**Moderate**: all the following (AND absence of criteria for more severe cases)	
1. Cough AND (sick appearing OR pneumonia at chest X-ray)	
**Severe**: any of the following (AND absence of criteria as for critical case)	
2. Oxygen saturation < 92%	
3. OR difficult breathing or other signs of severe respiratory distress (apnea, gasping, head nodding)	
4. OR need for any respiratory support	
**Critical**: Any of the following	
1. Patient in ICU	
2. OR intubated	
3. OR multiorgan failure	
4. OR shock, encephalopathy, myocardial injury or heart failure, coagulation dysfunction, acute kidney injury.	

Adapted from Dong Y et al. [4]

### Data analysis

Categorical variables were reported as absolute numbers and percentages and compared using the *χ*^2^, Fisher exact test, or Mantel-Haenszel correction as appropriate, and by calculating odds ratios (OR) with confidence intervals of 95% (95% CI). The significance level was set at 0.05 (two-tailed test). Continuous variables were expressed as means and standard deviations or as median and inter-quartile ranges (IQR), if not normally distributed. An exploratory subgroup analysis was performed on disease severity by age group. Data were analyzed with STATA 15.

## Results

**Fig. 1:**
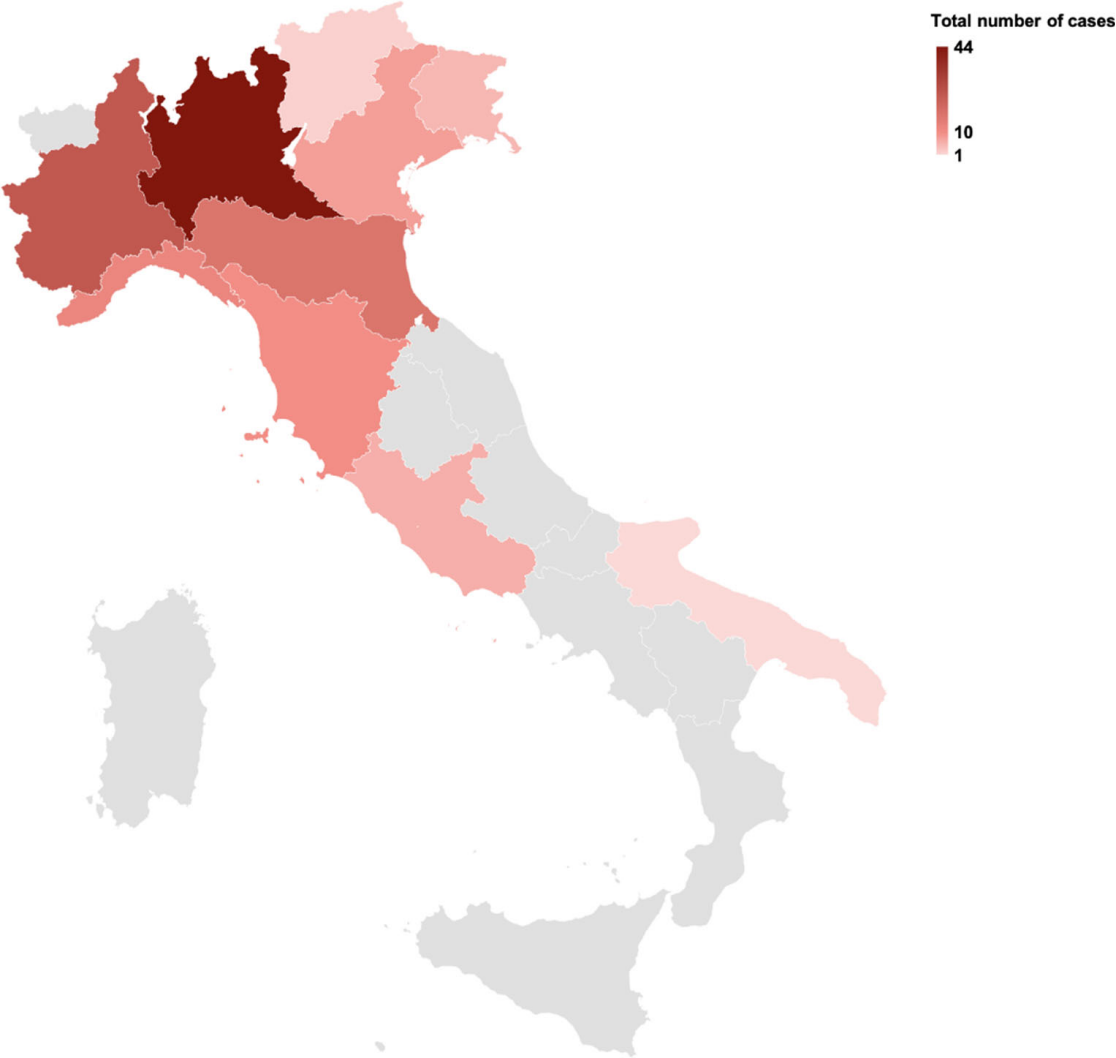
Distributions of enrolled COVID-19 cases across regions in Italy

Overall, 130 children and adolescents with confirmed COVID-19 virus infection were included in the study from 28 centers within the participating networks covering 10 regions in Italy (Fig. 1). One hundred twelve (86.2%) cases were recruited at hospital level, and 18 (13.8%) at outpatient level.

Notably, among patients younger than 2 years, 35/41 (85.3%) were less than 6 months of age (Table 2). Distribution by sex showed a slight male predominance (OR 1.63, 95% CI 1.00 to 2.68, *p* = 0.47). Overall, 70 (53.8%) of children had contact with a COVID-19 case, with most of these (67/70 (95.7%) reporting a relative with COVID-19. Thirty-four (26.2%) patients had comorbidities, with the most frequent being respiratory, cardiac, or neuromuscular chronic diseases (12% of all children).

**Table 2:** Sociodemographic characteristics and disease severity at presentation

	*N* = 130 *N* (%)	*p* values
Age range	0–17	NA
Median age (IQR)	6 (0–11)	NA
Age groups		
< 2 years	41 (31.5%)	*p* > 0.05
2–9 years	35 (26.9%)	
10–17 years	45 (34.6%)	
Missing	9 (6.9%)	
Sex		
Male	73 (56.2%)	*p* = 0.47
Female	57 (43.8%)	
Contact with COVID-19 positive	70 (53.8%)	NA
Relatives COVID-19 positive	67 (51.5%)	
Comorbidities		
Yes	34 (26.2%)	*p* < 0.001
No	92 (70.8%)	
Missing	4 (3.1%)	
Type of comorbidities		NA
Respiratory, cardiac or neuromuscular chronic diseases	16/130 (12.0%)	
Pre-term	3/130 (2.3%)	
Immunodepression	2/130 (1.4%)	
Cerebral palsy	1/130 (0.7%)	
Others^1^	12/34(10.0%)	
Disease severity		
Asymptomatic	17 (13.1%)	
Mild	81 (62.3%)	
Moderate	11 (8.5%)	*p* < 0.001
Severe	11 (8.5%)	
Critical	9 (6.9%)	
Missing	1 (0.8%)	
Symptoms and signs		NA
Fever	67/130 (51.5%)	
Dry cough	38/130 (29.2%)	
Productive cough	16/130 (12.3%)	
Rhinorrhea	25/130 (19.2%)	
Respiratory distress	17/130 (13.0%)	
Vomiting	15/130 (11.5%)	
Diarrhea	10/130 (7.6%)	
Sore throat	9/130 (6.9%)	
Thoracic pain	4/130 (3.0%)	
Hypo-reactive or hyperactive	4/130 (3.0%)	
Febrile convulsions	2/130 (1.5%)	
Otitis	1/130 (0.7%)	
Pains at lower limbs	1/130 (0.7%)	
Oxygen saturation level at presentation		NA
91–92	1/130 (0.8%)	
≤ 90	1/130 (0.8%)	
Laboratory test^1^	71/130 (54.6%)	NA
White blood cell count < 5.5 (× 10^9^/L)	7/19 (36.8%)	
Lymphocyte count < 1.2 (× 10^9^/L)	3/19 (15.7%)	
Aspartate aminotransferase > 50 (U/L; 10–50)	11/60 (18.3%)	
Alanine aminotransferase > 45 (U/L; 7–45)	8/68 (11.8%)	
Erythrocyte sedimentation rate > 20 mm/h	1/1 (100%)	
Chest X-ray ^1^	41/130 (31.5%)	NA
Ground-glass opacities	17/41 (41.5%)	
Negative	15/41 (36.6%)	
Focal consolidation	4/41 (9.8%)	
Missing description	5/41 (12.1%)	
Decision after first visit		NA
Discharged at home	55/130 (42.3%)	
Hospitalized	75/130 (57.7%)	
Respiratory support		NA
Oxygen	8/130 (6.1%)	
High flow oxygen	3/130 (2.3%)	
Non-invasive ventilation	2/130 (1.5%)	
Intubation	2/130 (1.5%)	
Cases in ICU	9/130 (6.9.0%)	
Outcome		NA
Cured	130/ (100%)	
Dead	0 (0%)	

*ICU* intensive care unit

^1^Other comorbidities: among these 12 cases, only the following were specified: anemia [2], thrombocytopenia, glucose-6-phosphate dehydrogenase deficiency (G6PDD) [1], nephritis [1], propionic acidemia [1], autism [1]

Most children were either asymptomatic (13.1%) or presented with mild disease (62.3%), while 11 (8.5%) had moderate disease, 11 (8.5%) had a severe disease, and 9 (6.9%) had a critical presentation.

Fever was recorded in 67 children (51.5%). The most common other symptom was cough, ether dry (29.2%) or productive (12.3%). Rhinorrhea was observed in 25 (19.2%). Respiratory distress was observed in 17 (13.0%). Two (1.6%) children were hypoxemic at presentation. Vomiting was reported in 15 (11.5%) and diarrhea in 10 (7.6%). Among children with vomiting, one had hematic vomit. Other signs or symptoms included sore throat (6.9%), thoracic pains (3%), hypo-reactivity (e.g., somnolence) or hyper-reactivity (e.g., excessive crying) (3%), febrile convulsions (1.5%), and pain in lower limbs (1.5%).

Out of the total sample of 130 children, 71 (54.6%) underwent laboratory testing. Among these patients, leucopenia and lymphopenia were detected in 36.8% and 15.7%, respectively, while increases in aspartate aminotransferase and alanine aminotransferase were reported in 18.3% and 11.8%, respectively.

Among the 41 (31.5%) children with chest X-ray, 17 (41.5%) showed ground-glass opacity, 15 (36.6%) presented a negative X-ray, and 4 (9.8%) had a focal consolidation.

Fifty-five (42.3%) children were treated at home and 75 (57.7%) were hospitalized.

Fifteen children needed some respiratory support: 8 (6.1%) needed oxygen, 3 (2.3%) high-flow oxygen, 2 (1.5%) non-invasive ventilation (CPAP), and 2 (1.5%) intubation and mechanical ventilation. Overall, nine (12.0%) children were admitted to intensive care unit (ICU).

Further characteristics of the children in the ICU are reported in Supplement 1. Out the nine cases in the ICU, six had an age below 6 months, and three were adolescents; seven were males. All children in the ICU were given some respiratory support, except for three cases, which were infants below 2 months of age (18, 31, and 41 days of life) with fever, and had either diarrhea, respiratory distress, or congenital conditions (anemia, congenital kidney malformation) plus a consolidation at chest X-ray. One adolescent with cerebral palsy, epilepsy tracheotomy, and enteral nutrition required mechanical ventilation. All children recovered, and none died.

Subgroup analysis (Table 3) revealed that children below 6 months of age had a significantly increased risk of “critical” disease severity when compared with older children (6/35 (17.1%) vs 3/86 (3.5%), two-tailed Fisher test *p* = 0.034, OR 5.6, 95% CI 1.3 to 29.1).

**Table 3:** Disease severity by age

	Asymptomatic	Mild	Moderate	Severe	Critical	Missing	Total
Age group							
< 6 months	2 (11.8%)	20 (24.7%)	4 (36.4%)	3 (30.0%)	*6 (60.0%)*	0 (0.0%)	35 (26.9%)
6–24 months	1 (5.9%)	4 (4.9%)	0 (0.0%)	1 (10.0%)	0 (0.0%)	0 (0.0%)	6 (4.6%)
2–9 years	7 (41.2%)	21 (25.9%)	4 (36.4%)	3 (27.3%)	0 (0.0%)	0 (0.0%)	35 (26.9%)
10–19 years	7 (41.2%)	27 (33.3%)	3 (27.3%)	4 (36.4%)	*3 (33.3%)*	*1 (100%)*	45 (34.6%)
Missing	0 (0.0%)	9 (11.1%)	0 (0.0%)	0 (0.0%)	0 (0.0%)	0 (0.0%)	9 (6.9%)
Total	17 (100%)	81 (100%)	11 (100%)	11 (100%)	9 (100%)	1 (100%)	130 (100)

Children below 6 months of age had a significantly increased risk of “critical” disease severity when compared with older children (6/35 (17.1%) vs 3/86 (3.5%)

## Discussion

This paper adds to previous knowledge on COVID-19 in children, describing the characteristics and outcomes of a sample of children diagnosed with the disease in Italy. Official national statistics in Italy, when the study recruitment ended, reported 704 cases of COVID-19 among patients below 20 years, accounting for 1% of total cases diagnosed country-wide [9]. The national surveillance system [9] recorded, at time of study end, only 49 cases of children with COVID-19 hospitalized, compared with the 75 hospitalized cases reported by our research networks and described in this study. Furthermore, national reports in Italy only provide a description of cases by age, and no further details on other children characteristics are available [9]. Major gaps in national surveillance data have been highlighted also in other countries, including the USA, with missing data on the variable of interest ranging from 9 to 91% of cases [7, 8]. This study, therefore, has the merit to identify a not negligible pediatric sample of COVID-19 cases in Italy, and characterized children by sociodemographic variables, comorbidities, severity of disease, clinical presentations, laboratory test, X-ray, and need of ventilatory support.

Case distribution across regions reflects voluntary participation of centers in the networks involved but is quite in line with the national distribution of cases of COVID-19, with Lombardy and Emilia-Romagna regions presenting the highest incidence of cases [9].

The hospitalization rate in the sample of this study was significantly higher than what is reported in the official [9] Italian statistics (57.7% vs 11.0%, *p* < 0.05). Also, 26.2% of children in the sample of this study had comorbidities, a rate which is likely to be higher than the expected within the general pediatric population. Based on these observations, we believe that our sample is biased toward a more fragile population with more severe presentation, consistently with a network mostly including hospitals. When making comparison across different studies on pediatric COVID-19 case series, it is important to acknowledge differences in the characteristics of the sample and enrollment site [4, 8, 11]. Specifically, in the largest study from China, most cases were diagnosed outpatient, and only 34.1% of cases were laboratory-confirmed [4]. Conversely, the only existing reports on children from Spain [5] are similar to our study, in the sense that children were mostly enrolled at hospital level; not surprisingly, hospitalization rate (60%) was similar to the rate observed in our study (57.7%). Early reports from the USA are difficult to interpret given the very high number of missing information [7]. When comparing across populations, it is critical to remember that, so far, the real number of COVID-19 virus–positive cases in each of the countries of the world is currently unknown, and most probably heavily underestimated [12]. Testing strategies and availability of diagnostic tests are largely variable across the globe, with Italy being among one of the countries with more test being performed, per million people [13]. Additionally, the validity of the diagnostic test currently used (PCR on nasal or pharyngeal swab) is subject of debate [14]. Therefore, the real incidence of COVID-19 severe and critical cases among the overall population, as well as the real hospitalization rate and the rate of cases in the ICU, is currently impossible to establish.

Results of this study confirm that COVID-19 in children is mostly a mild disease, however may have a not negligible rate of severe presentation in selected population of pediatric patients. Infants aged less than 6 months, especially males, seem significantly more susceptible to severe forms of the disease, in line with the previous Chinese case series [4]. Specific risk factors, including specific underlying diseases, for hospitalization and treatment in ICU in children are currently poorly described. Evidence need to be generated to further establish the incidence of severe presentation of COVID-19 in infants and in children with pre-existing diseases. Additionally, criteria for hospitalization and for admission in ICU, which may vary by setting, should be further documented.

When compared with existing literature, this case series identifies few novel presentations of COVID-19 in children, including thoracic pain, hypo-reactivity or hyper-reactivity, febrile convulsions, and pain in lower limbs. Other possible rare manifestations of the diseases in children, such as liver and heart injury [15], or skin rash [16], or isolated gastrointestinal symptoms [17], have been reported anecdotally and warrant further investigation.

## Electronic supplementary material

ESM 1 (DOCX 27.2 kb)

## Abbreviations

ARDS: Acute respiratory distress syndrome
ED: Emergency department
ICU: Intensive care unit
