# Supplementary material for: Characteristic of COVID-19 infection in pediatric patients: early findings from two Italian Pediatric Research Networks
Source: Eur J Pediatr. 2020 Jun 3;179(8):1315–23. doi: 10.1007/s00431-020-03683-8 (PMC7269687; doi:10.1007/s00431-020-03683-8)
Supplement: Supplementary file 1 — (DOCX 27.2 kb) [file 431_2020_3683_MOESM1_ESM.docx]

**SUPPLEMENT 1**

**Characteristic of COVID-19 infection in pediatric patients: Early findings from two Italian Pediatric Research Networks**

**Content**

Supplementary Table 1. Characteristics of children with COVID-19 in ICU page 2

**Supplementary Table 1. Characteristics of children with COVID-19 in ICU**

|  | N=9 |
| --- | --- |
| **Age group** |  |
| < 1 month | 3 (33.3%) |
| 2- 5 months | 3 (33.3%) |
| 6 months-9 years | 0 |
| 10-19 years | 3 (33.3%) |
| **Sex** |  |
| Male | 7 (77.8%) |
| Female | 2 (22.2%) |
| **City** |  |
| Brescia | 4 (44.4%) |
| Milano | 3 (33.3%) |
| Bergamo | 1 (11.1%) |
| Rovereto | 1 (11.1%) |
| **Contact with COVID-19 positive** | 2 (22.2%) |
| **Relatives COVID-19 positive** | 2 (22.2%) |
| **Co-morbidities** |  |
| Yes ^1^ | 3 (33.3%) |
| No | 5 (55.6%) |
| Missing | 1 (11.1%) |
| **Chest X-ray** |  |
| Ground glass opacities | 4 (44.4%) |
| Focal consolidation | 4 (44.4%) |
| Missing | 1 (11%) |
| **Key symptoms at presentation** |  |
| Fever | 6 (66.7%) |
| Respiratory distress | 3 (33.3%) |
| Cough | 3 (33.3%) |
| Diarrhea | 2 (22.2%) |
| **Respiratory support** |  |
| Oxygen | 3 (33.3%) |
| High flow oxygen | 1 (11.1%) |
| Noninvasive ventilation | 2 (22.2%) |
| Intubation | 1 (11.1%) |
| None ^2^ | 3 (33.3%) |

^1^ Comorbidities: anemia and congenital kidney malformation; congenital heart defect; cerebral palsy, epilepsy, tracheotomy, and enteral nutrition.

^2^ Characteristics of children without respiratory support:

one infant of 18 days of life had fever and respiratory distress

another child with 31 days of age, with fever, and diarrhea,

41 days of life, fever, anemia and congenital kidney malformation, and pneumonia.
